# Air-Stable Ultrabright Inverted Organic Light-Emitting Devices with Metal Ion-Chelated Polymer Injection Layer

**DOI:** 10.1007/s40820-021-00745-w

**Published:** 2021-12-06

**Authors:** Shihao Liu, Chunxiu Zang, Jiaming Zhang, Shuang Tian, Yan Wu, Dong Shen, Letian Zhang, Wenfa Xie, Chun-Sing Lee

**Affiliations:** 1 State Key Laboratory of Integrated Optoelectronics, College of Electronics Science and Engineering, Jilin University, 2699 Qianjin Street, Changchun, 130012 People’s Republic of China; 2 Department of Chemistry, Center of Super-Diamond and Advanced Films (COSDAF), City University of Hong Kong, Kowloon, Hong Kong, People’s Republic of China

**Keywords:** Air stability, Ultrabright, Electron injection, Metal ion chelation, Inverted organic light-emitting device

## Abstract

Here, this work presents an air-stable ultrabright inverted organic light-emitting device (OLED) by using zinc ion-chelated polyethylenimine (PEI) as electron injection layer. The zinc chelation is demonstrated to increase the conductivity of the PEI by three orders of magnitude and passivate the polar amine groups. With these physicochemical properties, the inverted OLED shows a record-high external quantum efficiency of 10.0% at a high brightness of 45,610 cd m^−2^ and can deliver a maximum brightness of 121,865 cd m^−2^. Besides, the inverted OLED is also demonstrated to possess an excellent air stability (humidity, 35%) with a half-brightness operating time of 541 h @ 1000 cd m^−2^ without any protection nor encapsulation.

**Supplementary Information:**

The online version contains supplementary material available at 10.1007/s40820-021-00745-w.

## Highlights

- By using a zinc ion-chelated polyethylenimine as electron injection layer, an inverted organic light-emitting device (OLED) was successfully prepared with an external quantum efficiency over 10% at a brightness of 45,610 cd m^−2^.
- The chelation between zinc ion and amine groups not only passivates polar amine groups, but also fuses separated electronic orbitals.
- The inverted OLED show an excellent air stability with an operating time of 541 h @ 1,000 cd m^−2^ without any protection.

**Supplementary Information:**

The online version contains supplementary material available at 10.1007/s40820-021-00745-w.

## Graphical abstract

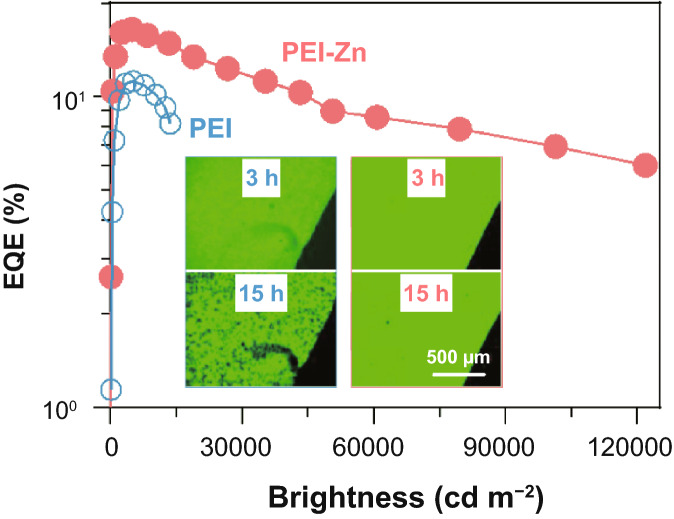

## Introduction

Organic light-emitting device (OLED) displays have gained momentum as displays for TV and smartphone in the past ten years [1]. With time goes on, information displays are no longer limited to flat panels that are just placed in front of the users but aimed at revolutionizing the way of interactions between the users and their surrounding environment [2–4]. Many new potential applications would demand high performance at high brightness. In particular, a brightness over 10,000 cd m^−2^ is needed for see-through display (such as Google glass), augmented reality (AR) display and high-dynamic range (HDR) display [2]. So far, most efforts have been made to achieve the theoretical maximum efficiency of OLEDs [5–8], whereas high-brightness performance is not getting as much attention. In order for OLEDs to be competitive with other light-emitting technologies, such as *µ*LED, high-brightness performance of OLEDs would have to be improved.

Moreover, reactive materials, such as alkali metals and salts, can be not used in inverted OLEDs with a bottom cathode. Inverted OLEDs are thus considered as an ideal structure for constructing air-stable OLEDs [9–13]. For example, it has been demonstrated that with a same encapsulation, a flexible display using inverted OLEDs can work after over 1 year, while that using conventional OLEDs cannot work normally after 21 days [13]. Due to high injection barrier (over 1 eV) from the metal oxides cathode to the organic layer, an electron injection interlayer (EJL) comprising amine-based molecules is used to promote electron injection in inverted OLEDs [12, 14–16]. In fact, inverted OLEDs with polyethylenimine (PEI) interlayer have achieved driving voltages and efficiencies comparable to the conventional devices [15]. However, high-brightness performances of OLEDs are limited by conductive properties of the amine-based polymers. It has to be noted that while most typical organic semiconductors have energy gap (*E*_g_) around 3 eV, PEI is an insulator with an *E*_g_ over 5 eV [16]. At high voltages, the PEI will become the main hurdle for improving high-brightness performance. As a result, ultrabright inverted OLEDs (over 100,000 cd m^−2^) have rarely been reported so far.

Here, we report an air-stable ultrabright inverted OLEDs by using zinc ion-chelated PEI (PEI-Zn) as EJL. The chelation between zinc ion and amine group can fuse separated orbitals of two neighboring PEI chains into a molecular orbital. The conductivity of the Zn-chelated PEI is nearly three orders of magnitude higher than that of PEI. Besides, the chelation of zinc ions also reduces the chemical reactivity of the amine groups and thus suppresses the degeneration effect of PEI. Finally, an ultrabright inverted OLED with a record-high maximum brightness of 121,865 cd/m^2^ is prepared by using PEI-Zn as EJL. The inverted OLED still shows an external quantum efficiency (EQE) over 10% at a high brightness of 45,000 cd m^−2^. Interestingly, without any protection nor encapsulation, the inverted OLED with PEI-Zn also shows a half-brightness operating time of 541 h @ 1000 cd m^−2^ in air (humidity, 35%).

## Methods

### Materials and Device Fabrication

Polyethylenimine (PEI, Mw: ~ 10,000) and zinc acetate dihydrate were purchased from Shanghai Aladdin Biochemical Technology Co. Ltd. MoO_3_, 4,4ʹ-N,Nʹ-dicarbazole (CBP), di-[4-(N,N-di(p-tolyl)-amino)-phenyl] cyclohexane (TAPC), 4,4',4"-Tris(carbazol-9-yl) triphenylamine (TCTA), 2,2ʹ,2ʹʹ-(1,3,5-benzinetriyl)-tris(1-phenyl-1-H-benzimidazole) (TPBi) and tris (2-phenylpyridine) iridium (III) (Ir(ppy)_3_) were obtained from Luminescence Technology Corporation. 10,10ʹ-[5-(6-[1,1ʹ-Biphenyl]-4-yl-2-phenyl-4-pyrimidinyl)-1,3-phenylene] bis [9,10-dihydro-9,9-dimethyl-acridine] (DMAC-BPP) was synthesized by Beijing Tuocai Optoelectronics Technology Co. Ltd. All materials and solvents were used as received without further purification. To prepare PEI-Zn precursors, 0.0075 g zinc acetate dihydrate was added to the 1 mL PEI (0.1 wt%, 2-methoxyethanol) solution and stirred until the solution became clear. A small amount (40 μL) of water was added to facilitate dissolving the zinc acetate dihydrate. After that, the white PEI-Zn polymer was obtained by freeze-drying in reserve. Before devices fabrication, the ITO glass substrates were cleaned with Decon 90, ultrasonic cleaning in deionized water and dried in the oven. Then, the substrates were processed in a plasma cleaner chamber (PDC-32G, Harrick). The PEI solution or PEI-Zn precursor solution was spin-coated on substrates at 3000 rpm for 40 s, following a thermal annealing at 150 °C for 10 min. Organic layers and anode were deposited sequentially by vacuum thermal evaporation process on the substrates under vacuum (~ 6 × 10^–4^ Pa). The deposition rate of organic layers, MoO_3_ and Ag anode is 0.3–1, 0.1–0.15, and 1.5–2.5 Å s^−1^, respectively.

### Film and Device Characterizations

Current density–voltage-luminance characteristics and EL spectra of unpackaged devices without any light outcoupling instruments were measured with a Goniophotometric Measurement System based on spectrometer (GP-500, Otsuka Electronics Co., Osaka, Japan) in the air at room temperature simultaneously. Ultraviolet and X-ray photoelectron spectrometer measurements were carried out with a high-resolution photoemission spectrometer (R3000, PREVAC). Transient PL decay curves of the devices were measured using a IHR320 spectrometer (HORIBA, Japan). Transient EL measurements were carried out with an arbitrary waveform generator (Rigol, DG5102), and transient responses of the devices were obtained with a HOLITA fluorescence spectrum analyzer system (HOLITA Co. Beijing, China). Capacitance–voltage curves were obtained with an impedance analyzer (TH2829C, Tonghui Co., Changzhou, China). Operating lifetime of the unpackaged devices was measured by using an OLED aging tester (ZJZCL-1, Shanghai University, China) under a constant current in atmospheric conditions. Images of light-emitting areas of devices were obtained by fluorescence microscope (Mshot M53, Guangzhou, China). The FTIR spectra were measured by using a Nicolet iS50 FTIR Spectrometer. Electrochemical measurements were performed using an IVIUMnSTAT multichannel electrochemical analyzer. A three-electrode electrochemical cell consisted of a platinum-connected ITO electrode as the working electrode, an Ag/AgCl (vs. Ag/AgCl, 3 M KCl) as the reference electrode, and a platinum plate as the counter electrode. pH values were measured by using a pH meter (PH808, Smart Sensor, Dongguan, China).

## Results and Discussion

### Device Structure of Inverted OLED with PEI-Zn Electron Injection Layer

**Fig. 1:**
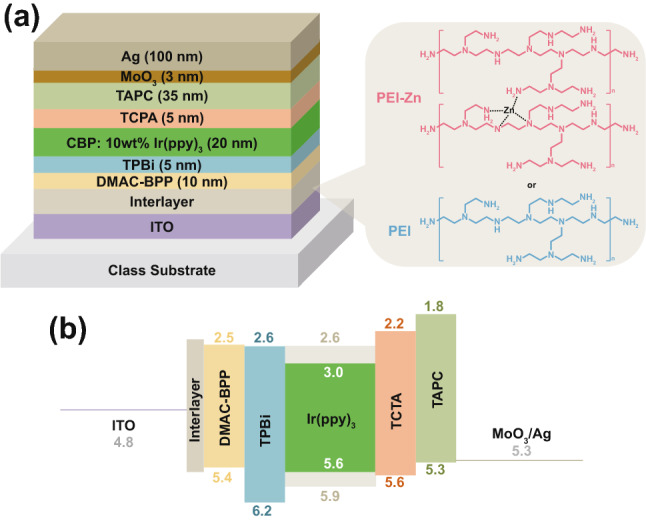
**a** Device structure and **b** energy level diagram of an inverted OLED with a PEI-Zn or a PEI interlayer

Figure 1 shows device structures and energy level diagrams of inverted OLEDs with a PEI-Zn EJL or with a PEI EJL. Molecular structures of the used small molecular materials in the inverted OLEDs are shown in Fig. S1. Here, an inert Ag layer modified by MoO_3_ is used as the top refractive anode, while an ITO is used as the bottom transparent cathode. The emitting layer consists of a bipolar host CBP doped with a green phosphor (Ir(ppy)_3_). The PEI-Zn or the PEI interlayer is deposited between the bottom ITO cathode and electron transport layer. With reference to the previous work, a 15-nm PEI or PEI-Zn interlayer is used in this work. It is because a thicker amine-containing polymer layer did not further reduce the working function and would adversely affect device performances due to its insulating natures [16]. The PEI-Zn in this work is synthesized by following the reported method [17], and the obtained sample is a white polymer (Fig. S2). Besides, effects of using different amount of zinc acetate dihydrate were also studied (Fig. S3). Compared to organic solar cells, active layers of OLEDs always have much deeper lowest unoccupied molecular orbital (LUMO) [18–20]. Thus, device performances of OLEDs are more sensitive to the working function of the interlayer. The 0.037 g of zinc acetate dihydrate in Zhou’s work will lead to a working function of 4.1 eV [17], and thus poor electron injection in OLEDs (see Fig. S3). The amount of zinc acetate dihydrate was thus reduced to 0.0037 g to ensure efficient electron injection and enhanced conductivity. With these parameters, efficient inverted OLEDs were prepared in this work.

### Characteristics of PEI-Zn Film

**Fig. 2:**
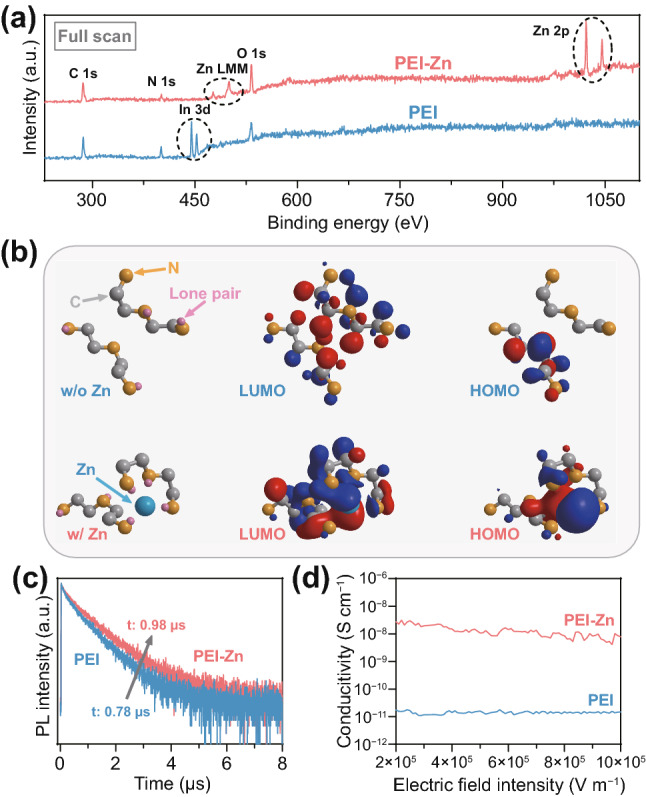
**a** XPS spectra of a PEI and a PEI-Zn layers coated on ITO substrates. **b** Optimized structure and molecular orbitals of two enamine molecules with and without zinc ions. **c** Transient PL characteristics (@520 nm) of green emitting layer (CBP:10 wt% Ir(ppy)_3_) deposited, respectively, on PEI and PEI-Zn. **d** Conductivity of PEI and PEI-Zn

Firstly, properties of the PEI-Zn coated on ITO are investigated as shown in Fig. 2 and S4-S9. Figure 2a shows X-ray photoelectron spectroscopy (XPS) results of a PEI-Zn and a PEI layer coated on ITO substrates. The Zn 2p (1021 and 1045 eV) and Zn LMM (476 and 500 eV) peaks can be clearly seen in the PEI-Zn sample. The N1s peak in PEI-Zn shifts to a higher binding energy (Fig. S4) compared to that of PEI. As shown in the Fourier transform infrared (FTIR) spectra (Fig. S5), the pristine PEI shows the stretching vibration of the -NH_2_ at 3360 cm^−1^ and the bending vibration of the -NH_2_ at 1664 cm^−1^, while the PEI-Zn does not. Besides, the -NH stretching vibration at 3285 cm^−1^ and bending vibration at 1600 cm^−1^ of the PEI are also found to shift to lower wavenumbers in the PEI-Zn. These results confirm the chelation between Zn ions and the polar amine groups (-NH_2_ and -NH) of the PEI chains. Interestingly, we also observe the In 3d (445 and 452 eV) peaks in PEI, but not in PEI-Zn (Fig. 2a). It is considered as a result of the passivated reactivity of the PEI-Zn compared to the PEI. As we also see from their 0.1 wt% aqueous solution, the pH (10.3) of the PEI solution is much higher than that (6.9) of the PEI-Zn solution. The absence of In 3d peaks in PEI-Zn indicates that the chelation between amine groups and Zn ions can avoid the corrosion to the ITO during the preparation of amine-based layers. Besides, following the method in the previous work [21], we measured the electrochemical stability of the PEI and the PEI-Zn films. As shown in Fig. S6, the PEI-Zn film also shows an enhanced electrochemical stability compared to the PEI film. The operating lifetime of inverted OLEDs with PEI-Zn is thus considered to benefit from these properties of the PEI-Zn, and the evidence is shown below in this paper.

The chelation between Zn ions and amine groups can also efficiently enhance the conductivity and passivate the polar amine groups of PEI (Fig. 2b–d). We take molecular mechanics calculations using a model of chained enamine molecule to represent the PEI chain for simplicity. The optimized structures (left of Fig. 2b) and the molecular orbitals (middle and right of Fig. 2b) of two enamine molecules with and without zinc ions are obtained from the theoretical calculations. We can see that after adding a zinc ion, some electron lone pairs of amine groups are attracted by the zinc ions, corresponding to the increase in the binding energy of the N 1 s (Fig. S4). It is reasonably considered that the rearrangement of the polar amine groups can obviously reduce the polarity of the PEI-Zn as the rearranged dipoles could cancel each other. Interestingly, the chelation effect also enhances the electronic coupling between the two enamine molecules. As we seen from Fig. 2b, there are only separated molecular orbitals in the two enamine molecules without zinc ion, while the molecular orbitals are combined into a fused orbital after adding a zinc ion. According to these theoretical results, the polarity and resistivity of the PEI-Zn should be both reduced compared to those of PEI due to the rearrangement of the polar amine groups and the orbitals fusion. To experimentally verify these, we deposited green organic emitting films (CBP:10 wt% Ir(ppy)_3_) on a PEI and a PEI-Zn films and measured their transient EL characteristics (Fig. 2c). Figure 2c shows that the exciton lifetime of the green emitter increases from 0.76 µs on the PEI to 0.98 µs on the PEI-Zn. In fact, the pristine exciton lifetime of the Ir(ppy)_3_ should be about 1 µs [22]. This indicates that the PEI has a serious quenching effect due to serious dipole–dipole interactions, while the PEI-Zn has negligible. The conductivities of the PEI and PEI-Zn are also measured and shown in Fig. 2d. The conductivities are obtained by measuring the voltage-current characteristics of a 15-nm-thick PEI or PEI-Zn on an ITO grid (Fig. S7). Because of the low working functions of PEI and PEI-Zn (Fig. S8), it can be reasonably considered that only electrons inject from ITO and transport through the PEI or the PEI-Zn films, and the conductivities of Fig. 2d belong to electron conductivities rather than the overall charge conductivities. As seen in Fig. 2d, the electron conductivity of the PEI-Zn is nearly three orders of magnitude higher than that of the PEI. Tauc plots (Fig. S9) also reveal a reduction of optical energy gap from 5.2 eV of the PEI to 3.6 eV of the PEI-Zn. The reduced energy gap of the PEI-Zn can lead to an increase in carrier density. Moreover, combined the fact that the structural building blocks of PEI chains are held together by comparatively weak van der Waals forces, the chelation can also enhance carrier mobility with the fused molecular orbital (Fig. 2b). Considering the above, the enhanced conductivity of the PEI-Zn is a result of the combination of its enhanced carrier density and mobility. Nevertheless, because the pristine PEI has a high cationic charge-density [16], it is reasonable to consider the enhanced carrier mobility as the major reason of the enhanced conductivity of the PEI-Zn. It should also be noted that the passivation and the electronic coupling of the PEI chains are not achieved by sacrificing their electron injection ability. As we see from the photoemission cutoff obtained via UPS spectra (Fig. S8), the working function varies by less than 0.1 eV before and after adding the zinc ions.

### Performances of OLED with PEI-Zn EJL

**Fig. 3:**
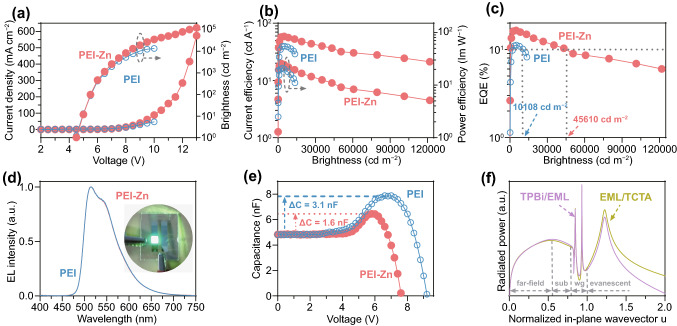
**a** Current density–voltage–brightness, **b** current efficiency–brightness–power efficiency, **c** EQE–brightness, **d** EL spectra and **e** capacitance–voltage characteristics of device PEI and device PEI-Zn, **f** power dissipation spectra of dipole sources at the EML/TCTA interface and the TPBi/EML interface

Two inverted OLEDs (Fig. 1a, b) using PEI or PEI-Zn as EJL are, respectively, prepared and marked as device PEI and device PEI-Zn. Current density–voltage–brightness, current efficiency–brightness–power efficiency, and EQE–brightness characteristics of the two devices are shown in Fig. 3a–c. Figure 3a shows that the two devices show similar current density–voltage characteristics below 8 V, while they behave differently at higher voltages. Device PEI-Zn shows much higher current density at the voltage over 8 V, and even works well after device PEI breaks down (@10 V). Due to their current density–voltage characteristics, device PEI shows a maximum brightness of about 15,000 cd m^−2^, which is at the same level with other reported inverted OLEDs [9, 10, 12, 13, 23–30], but device PEI-Zn shows a record-high brightness of 121,865 cd m^−2^. Besides, as shown in Fig. 3b, c, device PEI-Zn also has higher efficiencies than device PEI across the whole brightness range. For example, at the brightness of 5000 cd m^−2^, the EQE of device PEI-Zn is 16.4%, while that of device PEI is only 11.1%. More interestingly, the maximum brightness with a EQE over 10% can attain to 45,610 cd m^−2^ for device PEI-Zn, which is nearly 4.5 times of that (10,108 cd m^−2^) for device PEI. For comparison, device ZnO/PEI with ZnO (30 nm)/PEI (15 nm) as electron transport layer is also prepared (Fig. S10). Device performances of the inverted OLEDs of this work and the recently reported devices are summarized in Table 1. Compared to device PEI and device PEI-Zn, device ZnO/PEI shows a higher current density and thus a lower turn-on voltage (@ 2.7 V). The enhanced carrier injection of device ZnO/PEI is attributed to the reduced working function of ZnO-coated ITO (4.5 eV) [31]. Besides, device ZnO/PEI shows a maximum brightness of about 38,932 cd m^−2^, which is two times higher than that (~ 15,000 cd m^−2^) of device PEI but still far lower than that (121,865 cd m^−2^) of device PEI-Zn. In addition, the maximum efficiencies of device ZnO/PEI are inferior to those of device PEI and device PEI-Zn (see Table S1 and the reason is discussed in the later). As noted above, device PEI-Zn shows much better high-brightness performances compared to device PEI, device ZnO/PEI and previously reported inverted OLEDs [9, 10, 12, 13, 23–30].

**Table 1:** Device performances of inverted OLEDs

ETL	V_T_^a^ (V)	Maximum	@10,000 cd m^−2^					
		Brightness (cd/m^2^)	CE ^b^ (cd/A)	PE ^c^ (lm/W)	EQE (%)	CE (cd/A)	PE (lm/W)	EQE (%)
PEI-Zn (this work)	4.5	121,866	58.6	25.8	16.5	55.3	18.0	15.5
PEI (this work)	4.5	13,566	39.8	16.4	11.2	33.3	12.6	10.5
ZnO/PEI (this work)	2.6	38,932	31.1	19.5	9.1	23.7	10.0	6.6
ZnO/Bphen:CsCO_3_ (Ref. [23])	3.4	~ 4500	52.5	N/A	14.3	33.4	N/A	9.3
ZnO/s-SPB + N-DMBI (Ref. [26])	3.0	~ 4000	N/A	N/A	~ 15	N/A	N/A	N/A
ZnO/PEI /Cs_2_CO_3_:Alq_3_ (Ref. [10])	N/A	~ 35,000	53.3	40.8	19.7	~ 20.0	~ 10.0	~ 11.0
Mg doped ZnO/PEIE:Rb_2_CO_3_ (Ref. [30])	3.6	31,540	65.5	43.0	N/A	57.7	27.8	N/A

^a^The voltage at the brightness of 1 cd m^–2^

^b^Current efficiency

^c^Power efficiency

Considering their EL spectra (Fig. 3d) and atomic force microscope images (Fig. S11), the high-brightness performances of device PEI-Zn are not due to spectral nor morphological variations. We attribute the high-brightness performances to the enhanced conductivity of the PEI-Zn (Fig. 2d). Firstly, as shown in Fig. S12a, the resistance of a conventional OLED (ITO/MoO_3_ (3 nm)/TAPC (30 nm)/TCTA (5 nm)/CBP: 10 wt% Ir(ppy)_3_ (30 nm)/TmPyPB (50 nm)/LiF/Mg: 10 wt% Ag (120 nm)) decreases from 10^8^ to 10^2^ Ω as the bias voltage increases from 0 to 12 V. We also measured the resistances (Fig. S12b) of the PEI and the PEI interlayers by preparing electron-only devices with a structure of ITO/PEI or PEI-Zn/DMAC-BP (50 nm)/Mg: 10 wt% Ag (120 nm). Figure S12b shows that even at voltage ranging from 7 to 12 V, the PEI still has a high resistance of 10^6^ ~ 10^8^ Ω. Considering series circuit characteristics, device performances at high voltage would be seriously affected by the insulting property of the PEI layer (Fig. S12b). The enhanced conductivity (Figs. 2d and S12b) of the PEI-Zn is thus considered as a major reason for the performance improvements of device PEI-Zn at high voltage. It is found that the use of PEI-Zn can reduce the carrier accumulation inside the device. Figure 3e shows capacitance–voltage characteristics of the two devices. Device PEI has a 3.2 nF capacitance increase compared to its geometric capacitance, while that for device PEI-Zn is only 1.6 nF. With the parameters of Table S1, we simulated the hole accumulations in device PEI and device PEI-Zn. It can be seen in Fig. S13 that the higher capacitance increase of device PEI is due to hole accumulation at the EML/TPBI and the PEI/DMAC-BPP interfaces. The accumulated holes are considered to form a built-in field across the TPBi/DMAC-BPP/PEI to enhance the electron transport of PEI layer such that device PEI still shows voltage-current density characteristics similar to device PEI-Zn at the low voltage (< 8 V, Fig. 3a). However, it also leads to that the PEI sustains a much higher electric field and has a lower breakdown threshold (@10 V, Fig. 3a). Interestingly, it shows that device ZnO/PEI also breaks down at 10 V (Fig. S10). It supports that the PEI plays a key role in the breakdown threshold of OLEDs.

Secondly, the enhanced conductivity also allows the excitons formed at the TPBi/emitting layer (EML) interface not to be seriously quenched by triplet-polaron annihilation. We calculated the power dissipation spectra (Fig. 3f) of dipole sources at the EML/TCTA interface or the TPBi/EML interface with a homemade classic electromagnetic simulation software [32]. As shown in Fig. 3f, the peak at *u* = 1.22 can be clearly attributed to surface plasmon polaritons (SPPs) at the TAPC/anode interface. Coupling of dipole radiation to the SPP mode is much weaker at the TPBi/EML interface than at the EML/TCTA interface. It leads to a much higher outcoupling efficiency (21%) of excitons at the TPBi/EML interface compared to excitons (15%) at the EML/TCTA interface. The CBP with a high hole mobility is thus used as the host material in this work. Besides, the reason device ZnO/PEI has a lower efficiency due to the introduction of 30-nm ZnO which changes the waveguide modes (Fig. S10d) and leads to a lower outcoupling efficiency (~ 17%).

**Fig. 4:**
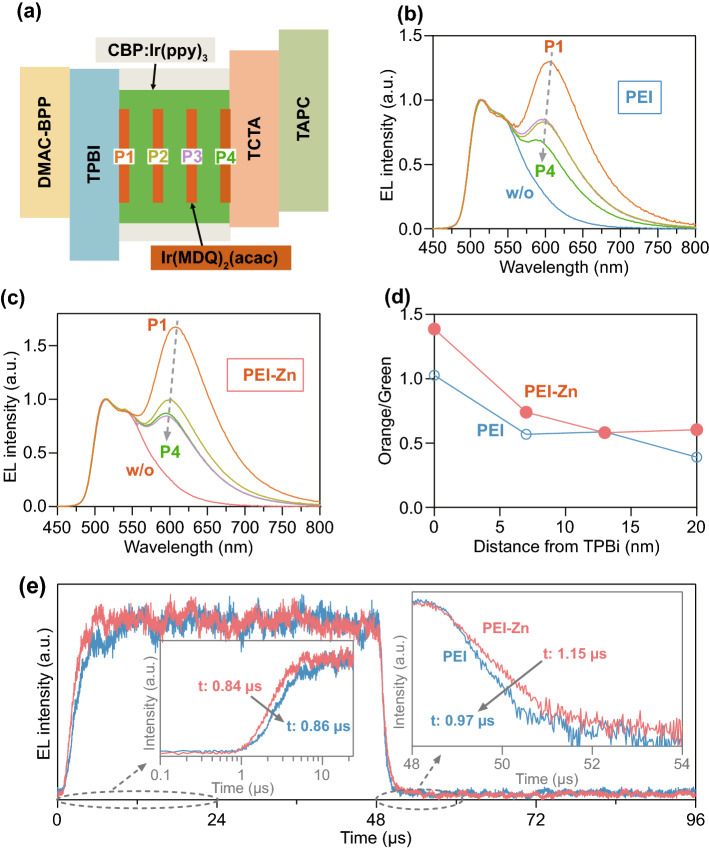
**a** Schematic diagram of devices (*P*1 to *P*4) with an orange probe at different distance from the TPBi/EML interface. EL spectra of **b** PEI-based devices and **c** PEI-Zn-based devices with the orange probe. **d** Ratios of orange emission to green emission for PEI-based devices and PEI-Zn-based devices with an orange probe. **e** Transient EL characteristics (@520 nm) of device PEI and device PEI-Zn without the orange probe

We then use a “probe detection” experiment to study the exciton distributions in the EMLs (Fig. 4a–c) and the influences of the EJLs on the exciton distributions (Fig. 4d, e). In the “probe detection” experiment, a 0.1-nm orange emitter Ir(MDQ)_2_(acac) is inserted in the EML as a probe. Four devices are, respectively, prepared by placing an orange probe at 0, 7, 13, and 20 nm from the TPBi/EML interface and marked as devices *P*1, *P*2, *P*3, and *P*4. Their EL spectra are then measured at the same current density of 3 mA cm^−2^, as shown in Fig. 4b, c. Figure S14 shows that an orange probe has little influence on carrier transport characteristics of the devices. Efficient energy transfers are also anticipated from CBP and Ir(ppy)_3_ to Ir(MDQ)_2_(acac). Thus, the ratios of orange emission to green emission can thus present the ratio of the excitons at/near the location of the orange probes to the whole excitons of the EML. The obtained exciton distributions of device PEI and device PEI-Zn are then shown in Fig. 4d. It shows that there is a dominant exciton distribution at the TPBi/EML interface for the two devices. However, the excitons of device PEI might be seriously quenched, as the excitons ratio of device PEI is much lower than device PEI-Zn at the TPBi/EML interface. The transient EL characteristics (Fig. 4e) of device PEI and device PEI-Zn are then measured by applying a transient voltage pulse (6 V @0 ~ 48 μs and 0 V @48 ~ 96 μs). It can be seen from Fig. 4e that device PEI-Zn shows a faster rise (left inset) and a slower decay (right inset) compared to device PEI. These results indicate excitons in device PEI-Zn suffer from a much weaker quenching effect compared to device PEI. Nevertheless, the CBP: Ir(ppy)_3_ films on PEI or PEI-Zn/DMAC-BPP/TPBI have same PL decay characteristics (Fig. S15). It indicates that the PEI or the PEI-Zn has no directly quenching effect on the excitons of emitting layer due to the DMAC-BPP/TPBI interlayer. According to the carrier accumulations (Fig. 3e), the quenching effect is considered to be related to the triplet-polaron annihilation (TPA). As shown in Fig. S13, a higher hole accumulation is anticipated at the EML/TPBI and the DMAC-BP/PEI interfaces of device PEI to form a built-in field and enhance the electron transport of the pristine PEI. Thus, more serious TPA is anticipated in device PEI and thus a slower rise and a faster decay.

**Fig. 5:**
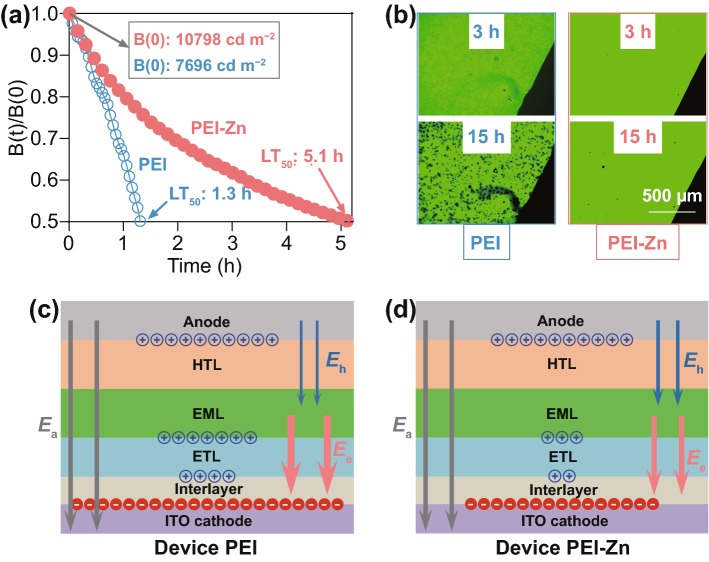
**a** Operating lifetimes of device PEI and device PEI-Zn at a current density of 20 mA cm^−2^. **b** Images of light-emitting areas of device PEI and device PEI-Zn after storing for 3 h and 15 h. Schematic mechanism of **c** device PEI and **d** device PEI-Zn

Moreover, due to the reduced carrier accumulation and the passivated amine groups (-NH_2_ and -NH), device PEI-Zn is also considered to show a better operating lifetime. Driven by a current density of 20 mA cm^−2^, the operating lifetimes of device PEI and device PEI-Zn are measured in air without any protection nor encapsulation. The initial brightness *B*(0) of device PEI and device PEI-Zn is 7,696 and 10,798 cd m^−2^, respectively. The L_50_ lifetime of an OLED is defined as the time it takes until its brightness reaches 50% of the initial value. As shown in Fig. 5a, device PEI-Zn has a L_50_ lifetime of 5.1 h for *B*(0) = 10,798 cd m^−2^, which is much higher than that (1.3 h for *B*(0) = 7696 cd/m^2^) of device PEI. The LT_50_ of device PEI-Zn for *B*(0) = 1000 cd m^−2^ is then extrapolated as 541 h by using a function LT_50_(L)/ LT_50_(H) = (H/L)^α^ with α = 1.96 [33]. It should be noted that the operating lifetime of device PEI-Zn is measured in air without any encapsulation. To our knowledge, the LT_50_ = 541 h for *B*(0) = 1,000 cd m^−2^ of OLEDs in air (humidity, 35%) without any encapsulation has never been reported. Besides, Fig. 5b shows images of the operating device PEI and device PEI-Zn after being stored for 3 and 15 h without any protection nor encapsulation. Due to the use of inert metal electrode, the inverted devices should exhibit much better air stability than conventional OLEDs. However, many non-emissive areas (black spots) with irregular shapes are found in device PEI. The black spots of device PEI are attributed to the loss of the ability of electron injection due to the slow reaction between PEI and organic semiconductors or ITO (Fig. 2a). On the other hand, there are only several small black spots with circle shape observed in device PEI-Zn. The regular black spot is known for researchers in the field of OLEDs as a result of the corrosion of water and oxygen to metal electrode [34, 35]. The much fewer black spots in device PEI-Zn clearly show the advantage of the use of an inverted structure and an inert metal electrode. Our results also indicate that the use of the passivated EJL with efficient electron injection/transport is a key requirement to take the advantage of inverted OLEDs.

### Working Mechanism for PEI-Zn EJL

As noted above, a mechanism of improvement in device PEI-Zn is proposed in Fig. 5c, d. The improvement of device PEI-Zn is related to a self-adapting behavior of OLEDs that enhances the balance of hole and electron currents. When the electron current is lower than the hole current, excess hole will accumulate at the interface between the EML and the electron transport layer (ETL) (Figs. 3e and S13) and affects internal electric field distribution. The increased electrical field across the ETL will then enhance the electron current to match the dominant hole current. In device PEI-Zn, the conductivity of PEI-Zn is much higher (Figs. 2d and S12b) such that a reduced carrier accumulation can satisfy the need of current balance. Due to the reduced carrier accumulation, enhanced device performances are anticipated in device PEI-Zn.

## Conclusions

In summary, an air-stable ultrabright inverted OLEDs is successfully prepared by using zinc ion-chelated PEI as electron injection layer. It demonstrates that the molecular orbitals of neighboring PEI chains can be fused together by the chelation between zinc ions and amine groups. It leads to two physicochemical properties of the zinc ion-chelated PEI: an enhanced conductivity and a passivated reactivity. Using the modified electron injection layer, air-stable ultrabright inverted OLEDs have been demonstrated with a record-high maximum brightness of 121,865 cd m^−2^ and an EQE over 10% at a brightness of 45,000 cd m^−2^. Interestingly, the inverted OLED can also show a record operating time of 541 h @ 1,000 cd m^−2^ in air (humidity, 35%) for unencapsulated devices. This work paves a way for constructing high-brightness and air-stable LEDs.

## Supplementary Information

Below is the link to the electronic supplementary material.

Supplementary file1 (PDF 797 kb)

## References

[1] Chen H-W, Lee J-H, Lin B-Y, Chen S, Wu S-T (2018). Liquid crystal display and organic light-emitting diode display: present status and future perspectives. Light Sci. Appl..

[2] Zhan T, Yin K, Xiong J, He Z, Wu S-T (2020). Augmented reality and virtual reality displays: perspectives and challenges. Iscience.

[3] Huang Y, Hsiang E-L, Deng M-Y, Wu S-T (2020). Mini-LED, micro-LED and OLED displays: present status and future perspectives. Light Sci. Appl..

[4] Kim H-M, Um JG, Lee S, Jeong DY, Jung Y (2018). High brightness active matrix micro-LEDs with LTPS TFT backplane. SID Symp. Dig. Tech. Pap..

[5] Sun Y, Forrest SR (2008). Enhanced light out-coupling of organic light-emitting devices using embedded low-index grids. Nat. Photonics.

[6] Liu S, Yu H, Zhang Q, Qin F, Zhang X (2019). Efficient ITO-free organic light-emitting devices with dual-functional PSS-rich PEDOT: PSS electrode by enhancing carrier balance. J. Mater. Chem. C.

[7] Chan C-Y, Tanaka M, Lee Y-T, Wong Y-W, Nakanotani H (2021). Stable pure-blue hyperfluorescence organic light-emitting diodes with high-efficiency and narrow emission. Nat. Photonics.

[8] Kim JU, Park IS, Chan C-Y, Tanaka M, Tsuchiya Y (2020). Nanosecond-time-scale delayed fluorescence molecule for deep-blue oleds with small efficiency rolloff. Nat. Commun..

[9] Höfle S, Schienle A, Bruns M, Lemmer U, Colsmann A (2014). Enhanced electron injection into inverted polymer light-emitting diodes by combined solution-processed zinc oxide/polyethylenimine interlayers. Adv. Mater..

[10] Zhao X-D, Li Y-Q, Xiang H-Y, Zhang Y-B, Chen J-D (2017). Efficient color-stable inverted white organic light-emitting diodes with outcoupling-enhanced ZnO layer. ACS Appl. Mater. Interfaces.

[11] Lee BR, Jung ED, Park JS, Nam YS, Min SH (2014). Highly efficient inverted polymer light-emitting diodes using surface modifications of ZnO layer. Nat. Commun..

[12] Dong WJ, Park JY, Ham J, Jung GH, Lee I (2016). Dual effect of ITO-interlayer on inverted top-illuminated polymer solar cells: wetting of polyelectrolyte and tuning of cavity. Adv. Funct. Mater..

[13] Fukagawa H, Sasaki T, Tsuzuki T, Nakajima Y, Takei T (2018). Long-lived flexible displays employing efficient and stable inverted organic light-emitting diodes. Adv. Mater..

[14] Lee BH, Jung IH, Woo HY, Shim HK, Kim G (2014). Multi-charged conjugated polyelectrolytes as a versatile work function modifier for organic electronic devices. Adv. Funct. Mater..

[15] Lee BR, Lee S, Park JH, Jung ED, Yu JC (2015). Amine-based interfacial molecules for inverted polymer-based optoelectronic devices. Adv. Mater..

[16] Zhou Y, Fuentes-Hernandez C, Shim J, Meyer J, Giordano AJ (2012). A universal method to produce low-work function electrodes for organic electronics. Science.

[17] Qin F, Wang W, Sun L, Jiang X, Hu L (2020). Robust metal ion-chelated polymer interfacial layer for ultraflexible non-fullerene organic solar cells. Nat. Commun..

[18] Wang M, Zhou L, Yu M, Liu C, Chu S (2017). Amphiphilic conjugated molecules with multifunctional properties as efficient blue emitters and cathode interlayers for inkjet printed organic light-emitting diodes. J. Mater. Chem. C.

[19] Gong Y, Zhang J, Du B, Wang M, Lai WY (2019). Design, synthesis, and postvapor treatment of neutral fulleropyrrolidine electron-collecting interlayers for high-efficiency inverted polymer solar cells. ACS Appl. Electron. Mater..

[20] Xu WD, Lai WY, Hu Q, Teng XY, Zhang XW (2014). A hydrophilic monodisperse conjugated starburst macromolecule with multidimensional topology as electron transport/injection layer for organic electronics. Polym. Chem..

[21] Miao Z, Wang X, Ma R, Zhu W, Li Y (2020). Dopamine semiquinone radical doped PEDOT: PSS: Enhanced conductivity, work function and performance in organic solar cells. Adv. Energy Mater..

[22] Song D, Zhao S, Aziz H (2011). Modification of exciton lifetime by the metal cathode in phosphorescent oleds, and implications on device efficiency and efficiency roll-off behavior. Adv. Funct. Mater..

[23] Chen Y, Chu S, Li R, Qin Y, Xu Y (2019). Highly efficient inverted organic light-emitting devices adopting solution-processed double electron-injection layers. Org. Electron..

[24] Xu W, Zhang X, Hu Q, Zhao L, Teng X (2014). Fluorene-based cathode interlayer polymers for high performance solution processed organic optoelectronic devices. Org. Electron..

[25] Fukagawa H, Morii K, Hasegawa M, Arimoto Y, Kamada T (2014). Highly efficient and air-stable inverted organic light-emitting diode composed of inert materials. Appl. Phys. Express..

[26] Fukagawa H, Hasegawa M, Morii K, Suzuki K, Sasaki T (2019). Universal strategy for efficient electron injection into organic semiconductors utilizing hydrogen bonds. Adv. Mater..

[27] Matsuo Y, Okada H, Kondo Y, Jeon I, Wang H (2018). Anthracene-based organic small-molecule electron-injecting material for inverted organic light-emitting diodes. ACS Appl. Mater. Interfaces.

[28] Ohisa S, Suzuki M, Chiba T, Kido J (2019). Doping of tetraalkylammonium salts in polyethylenimine ethoxylated for efficient electron injection layers in solution-processed organic light-emitting devices. ACS Appl. Mater. Interfaces.

[29] Chiba T, Pu Y-J, Ide T, Ohisa S, Fukuda H (2017). Addition of lithium 8-quinolate into polyethylenimine electron-injection layer in oleds: Not only reducing driving voltage but also improving device lifetime. ACS Appl. Mater. Interfaces.

[30] Kim J, Kim H-M, Jang J (2018). Low work function 2.81 eV Rb2CO3-doped polyethylenimine ethoxylated for inverted organic light-emitting diodes. ACS Appl. Mater. Interfaces.

[31] Yeh TC, Zhu Q, Buchholz DB, Martinson AB, Chang RPH (2015). Amorphous transparent conducting oxides in context: Work function survey, trends, and facile modification. Appl. Surf. Sci..

[32] Zang C, Liu S, Xu M, Wang R, Cao C (2021). Top-emitting thermally activated delayed fluorescence organic light-emitting devices with weak light-matter coupling. Light Sci. Appl..

[33] Yang Y, Zheng Y, Cao W, Titov A, Hyvonen J (2015). High-efficiency light-emitting devices based on quantum dots with tailored nanostructures. Nat. Photonics.

[34] Liu S, Zhang X, Wang S, Feng H, Zhang J (2018). Hybrid organic light-emitting device based on ultrasonic spray-coating molybdenum trioxide transport layer with low turn-on voltage, improved efficiency & stability. Org. Electron..

[35] van de Weijer P, Lu K, Janssen RR, de Winter SH, Akkerman HB (2016). Mechanism of the operational effect of black spot growth in OLEDs. Org. Electron..

