# Supplementary material for: Air-Stable Ultrabright Inverted Organic Light-Emitting Devices with Metal Ion-Chelated Polymer Injection Layer
Source: Nanomicro Lett. 2021 Dec 6;14:14. doi: 10.1007/s40820-021-00745-w (PMC8648931; doi:10.1007/s40820-021-00745-w)
Supplement: Supplementary file 1 — Supplementary file1 (PDF 797 kb) [file 40820_2021_745_MOESM1_ESM.pdf]

Supporting Information for

Air-Stable Ultrabright Inverted Organic Light-Emitting Devices with Metal Ion-Chelated Polymer Injection Layer

Shihao Liu[1, 2, #], Chunxiu Zang[1, #], Jiaming Zhang[1], Shuang Tian[2], Yan Wu[2], Dong Shen[2], Letian Zhang[1], Wenfa Xie[1, *], Chun-Sing Lee[2, *]

[1]State Key Laboratory of Integrated Optoelectronics, College of Electronics Science and Engineering, Jilin University, 2699 Qianjin Street, Changchun 130012, P. R. China

[2]Center of Super-Diamond and Advanced Films (COSDAF), Department of Chemistry, City University of Hong Kong, Kowloon, Hong Kong, P. R. China

[#]S.H. Liu and C.X. Zang have contributed equally to this work.

*Corresponding authors. E-mail: xiewf@jlu.edu.cn (Wenfa Xie), apcslee@cityu.edu.hk (Chun-Sing Lee)

Supplementary Figures and Table

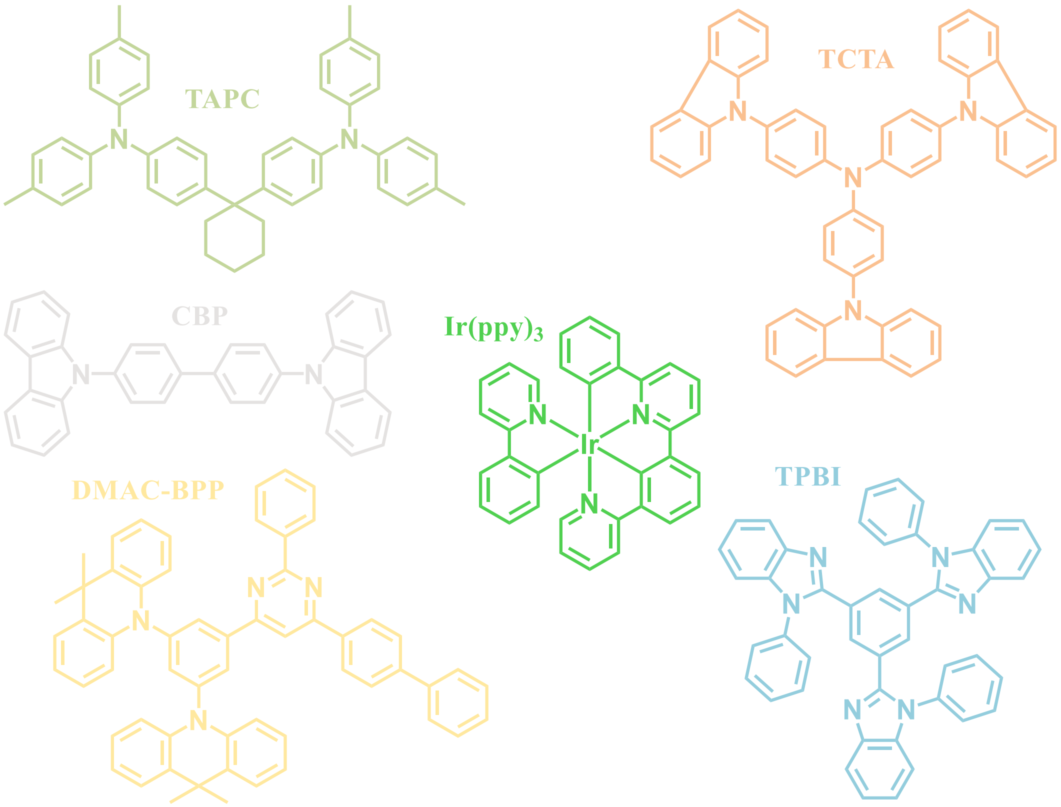

Fig. S1 Molecular structures of the used small molecular materials in the inverted OLEDs

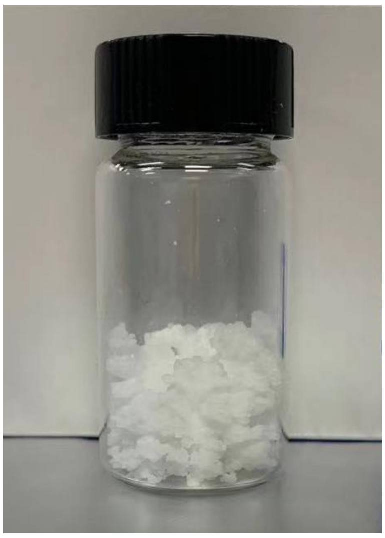

Fig. S2 A photograph of the synthesized PEI-Zn powder

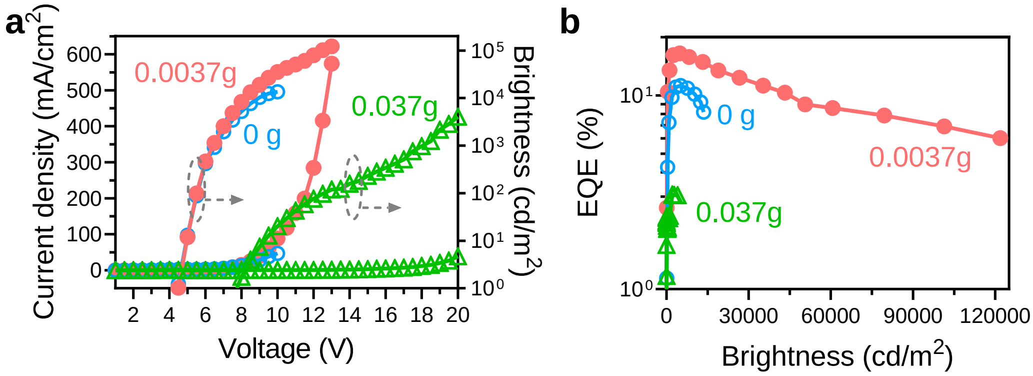

Fig. S3 (**a**) Current density-voltage-brightness and (**b**) EQE-brightness characteristics of device with PEI interlayer after adding zinc acetate dihydrate with different concentrations

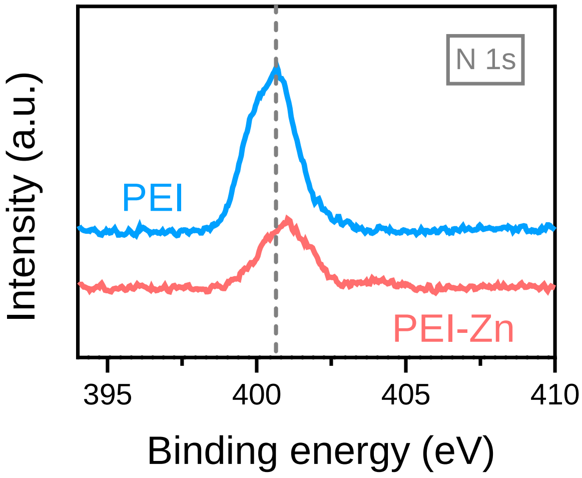

Fig. S4 N 1s XPS spectra of the PEI and the PEI-Zn layers coated on ITO

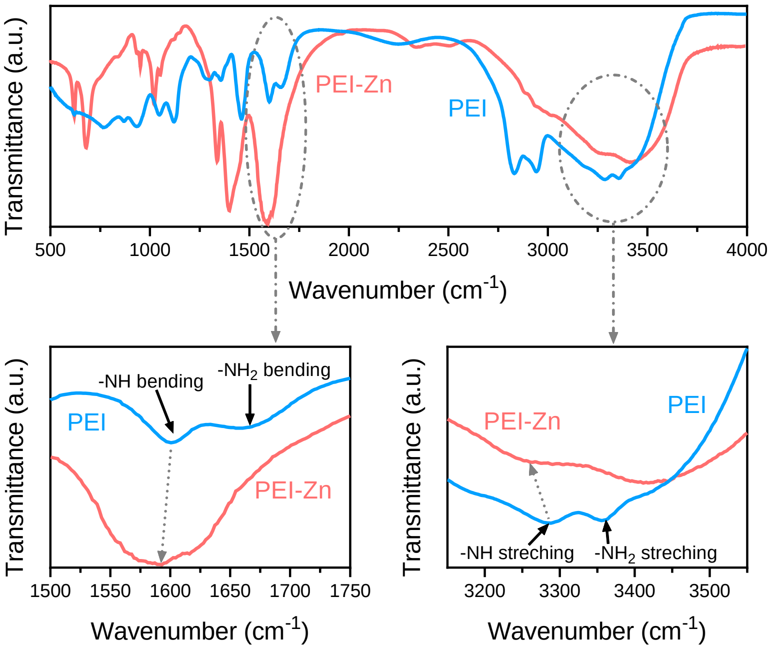

Fig. S5 FTIR spectra of the PEI and the PEI-Zn

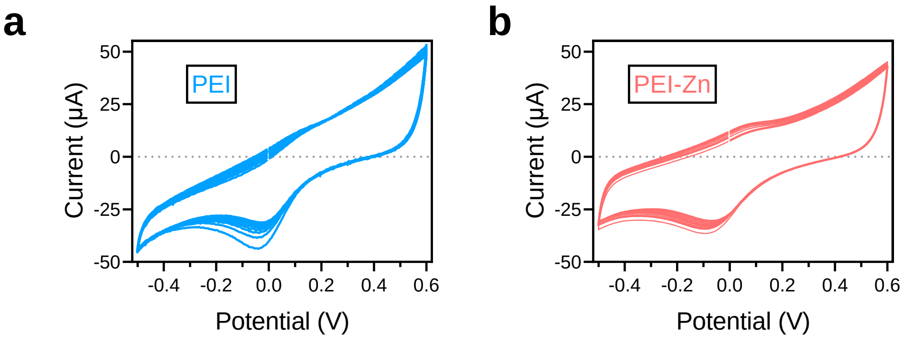

Fig. S6 Electrochemical stability of the PEI and the PEI-Zn films

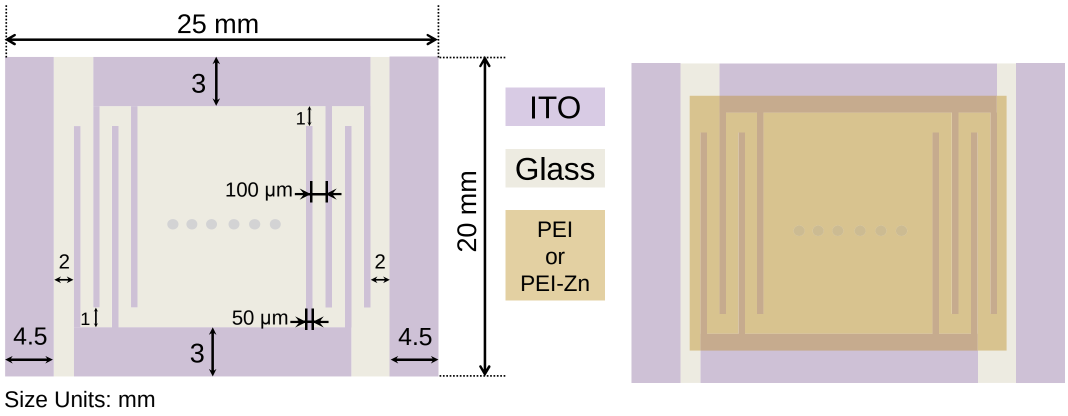

Fig. S7 Schematic diagram of the ITO grid substrate for the conductivity measurements

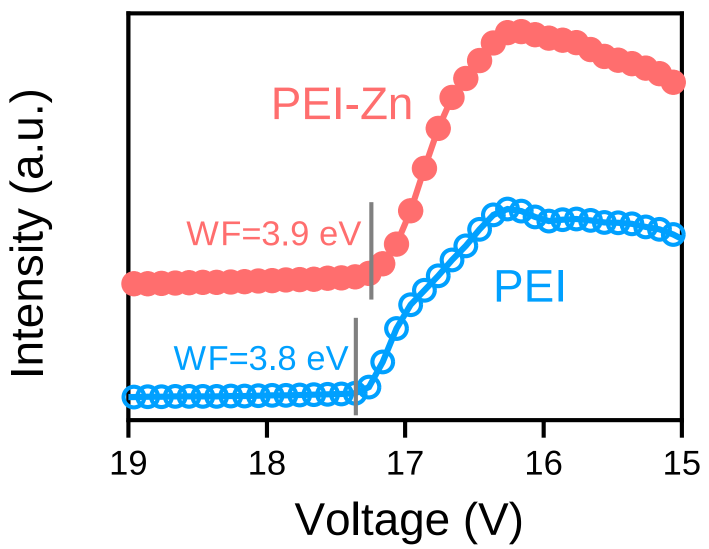

Fig. S8 UPS spectra of the PEI-Zn and the PEI coated on the ITO

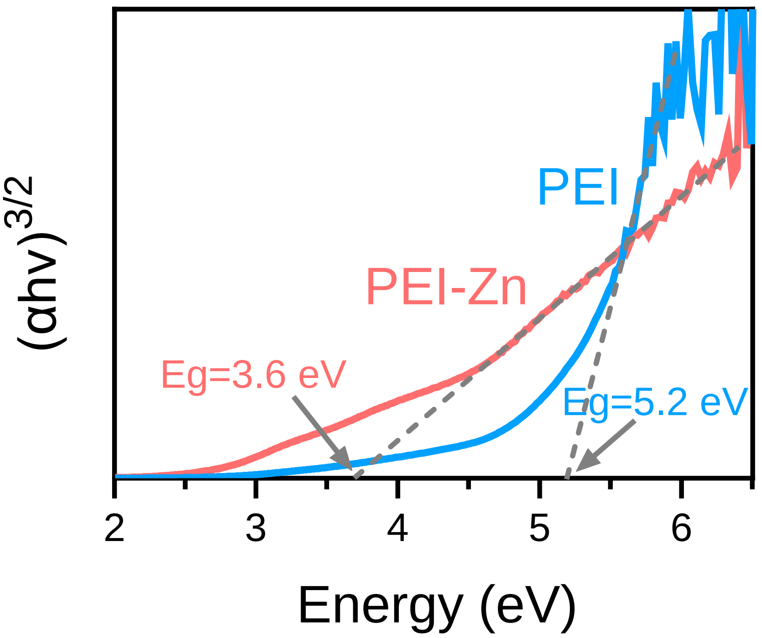

Fig. S9 Tauc plots of the PEI-Zn and the PEI

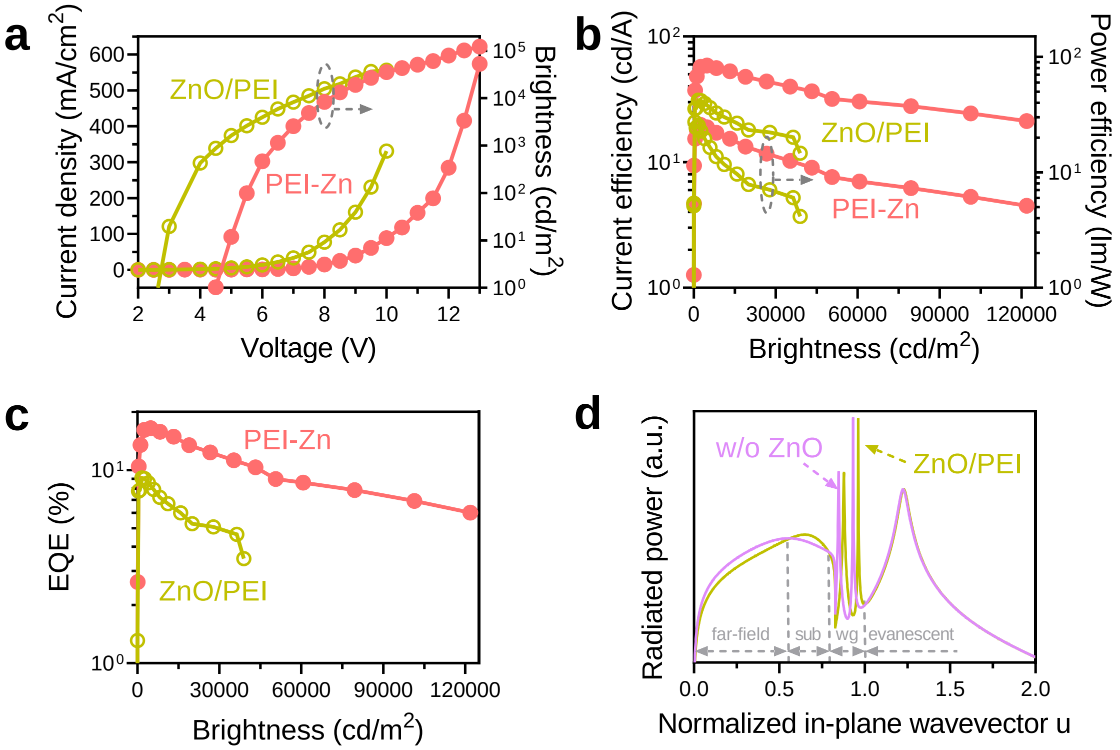

Fig. S10 (**a**) Current density-voltage-brightness, (**b**) current efficiency-brightness-power efficiency, (**c**) EQE-brightness and (d) optical power dissipation characteristics of device PEI-Zn and device ZnO/PEI. The structure of device Zn/PEI is ITO/ZnO (30 nm)/PEI (15 nm)/ DMAC-BPP (10 nm)/CBP: 10wt% Ir(ppy)$_3$ (20 nm)/TCTA (5 nm)/TAPC (35 nm)/MoO_3 (3 nm)/Ag (120 nm)

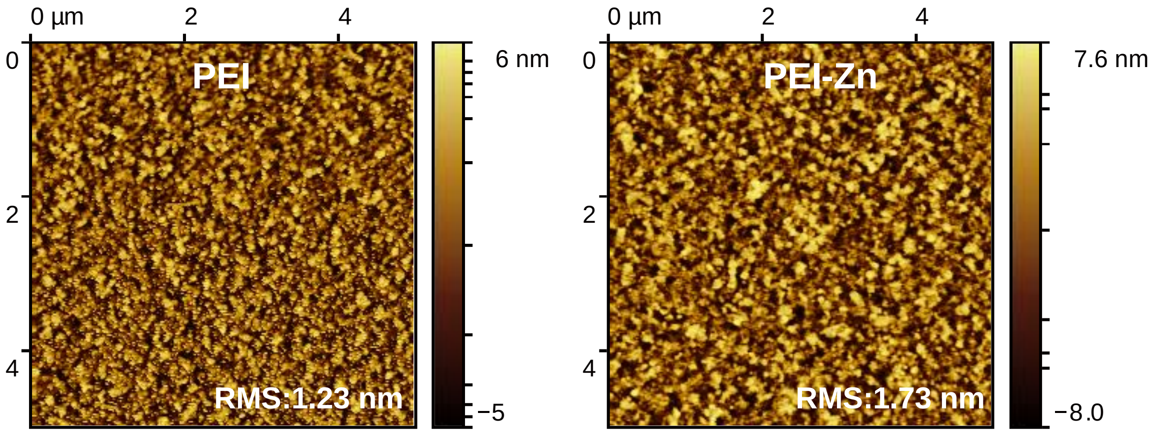

Fig. S11 Atom force microscope (AFM) images of PEI and PEI-Zn films

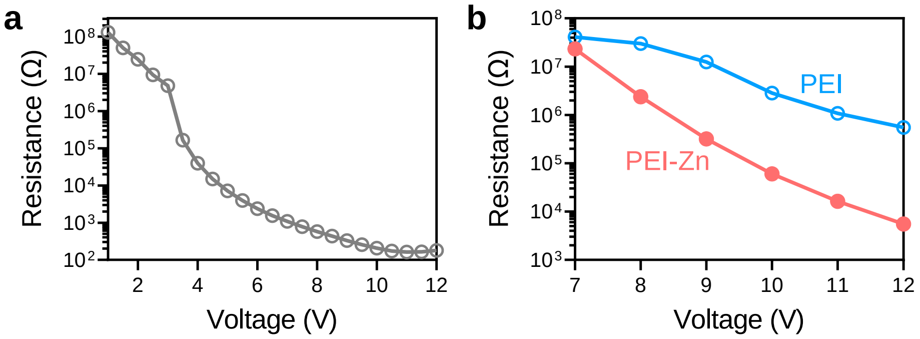

Fig. S12 (**a**) Resistance-voltage characteristic of a conventional OLED with a structure of ITO/MoO_3 (3 nm)/TAPC (30 nm)/TCTA (5 nm)/CBP: 10 wt% Ir(ppy)$_3$ (30 nm)/ TmPyPB (50 nm)/LiF/Mg: 10 wt% Ag (120 nm). (**b**) Resistance-voltage characteristics of PEI-based and PEI-Zn-based single carrier devices with a structure of ITO/PEI or PEI-Zn/DMAC-BP (50 nm)/Mg: 10 wt% Ag (120 nm)

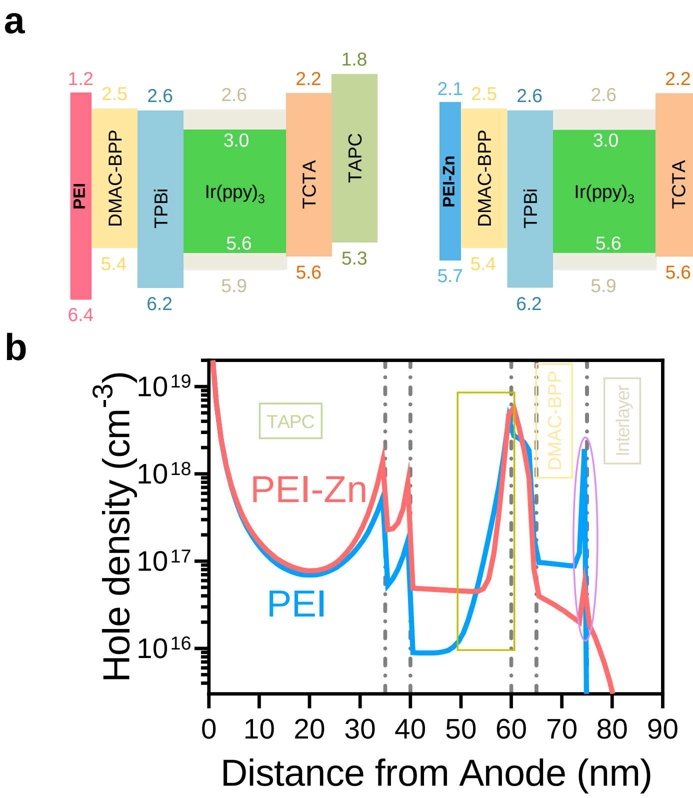

Fig. S13 (**a**) Energy levels of the PEI or the PEI-Zn used for the simulation of hole accumulations. (**b**) Simulated hole density of device PEI and device PEI-Zn. The energy levels of the PEI and PEI-Zn is calculated by following the method of Zhou et al (see Fig. S2 of ref. 22). The electron affinity is given by the equation of E_F+0.5*E_g, and the ionization potential energy is provided by the equation of E_F-0.5*E_g. The values of E_F and E_g are extracted from the Tauc plots (Figure S9) and the UPS spectra (Figure S8). The brown green rectangle and violet circle respectively represent the hole accumulation at the EML/TPBi interface and the DMAC-BPP/EJL interface. The simulation is conducted by the commercial simulation software SimOLED.

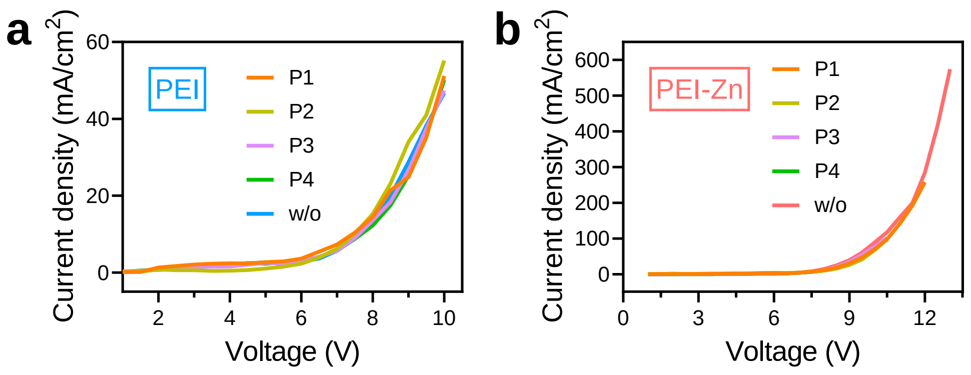

Fig. S14 Current density-voltage characteristics of (**a**) PEI-based devices and (**b**) PEI-Zn-based devices with an orange probe at different distance from the TPBi/EML interface

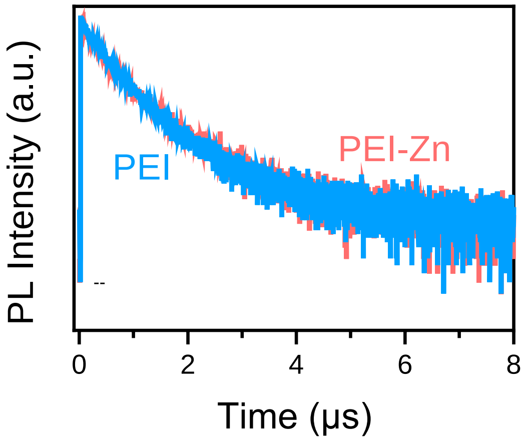

Fig. S15 Transient PL decay characteristics (@520 nm) of the CBP:10 wt% Ir(ppy)$_3$ films (20 nm) on the PEI/DMAC-BP/TPBi and the PEI-Zn/DMAC-BP/TPBI substrates

Table S1 Parameters for simulation of hole density

**Material**	**Mobilities (cm^2 V^{-1} S^{-1})**		**Energy Level (eV)**		**Relative dielectric constants**	**Thickness (nm)**
	Electron	Hole	LUMO	HOMO		
**ITO**	WF=-4.5				3.2	120
**PEI**	10^{-8}	10^{-8}	-1.2	-6.4	3.0	15
**PEI-Zn**	10^{-5}	10^{-5}	-2.1	-5.7	3.0	15
**DMAC-BPP**	8.5×10^{-5}	1.9×10^{-5}	-2.5	-5.4	3.0	10
**TPBI**	3.3×10^{-5}	10^{-6}	-2.6	-6.2	3.0	5
**CBP**	3×10^{-4}	10^{-3}	-2.6	-5.9	3.0	20
**Ir(ppy)$_3$**	10^{-6}	2.9×10^{-5}	-3.0	5.6	3.0	20, 10 wt%
**TCTA**	10^{-8}	3×10^{-4}	-2.2	-5.6	3.0	5
**TAPC**	10^{-6}	10^{-2}	-1.8	-5.3	3.0	35
**Ag**	WF=-5.3				-10.991+0.33i	100
